# Nurse navigation, symptom monitoring and exercise in vulnerable patients with lung cancer: feasibility of the NAVIGATE intervention

**DOI:** 10.1038/s41598-023-50161-w

**Published:** 2023-12-20

**Authors:** Rikke Langballe, Lukas Svendsen, Erik Jakobsen, Susanne Oksbjerg Dalton, Randi Valbjørn Karlsen, Maria Iachina, Karen M. Freund, Amy Leclair, Lars Bo Jørgensen, Søren T. Skou, Jeanette Haar Ehlers, Rikke Torenholt, Mads Nordahl Svendsen, Pernille Envold Bidstrup

**Affiliations:** 1 Psychological Aspects of Cancer, Cancer Survivorship, Danish Cancer Institute, Copenhagen, Denmark; 2 Department of Clinical Oncology and Palliative Care, Zealand University Hospital, Næstved, Denmark; 3 Department of Thoracic Surgery, Odense University Hospital, Odense, Denmark; 4 The Danish Lung Cancer Registry, Odense University Hospital, Odense, Denmark; 5 Cancer Survivorship, Danish Cancer Institute, Copenhagen, Denmark; 6 Institute of Clinical Medicine, Faculty of Health, University of Copenhagen, Copenhagen, Denmark; 7 Danish Research Center for Equality in Cancer, Department of Clinical Oncology & Palliative Care, Zealand University Hospital, Næstved, Denmark; 8 Center for Clinical Epidemiology and Research Unit of Clinical Epidemiology, Odense University Hospital, Odense, Denmark; 9 Department of Medicine, Tufts Medical Center, Tufts University School of Medicine, Boston, USA; 10 The Research and Implementation Unit PROgrez, Department of Physiotherapy and Occupational Therapy, Næstved-Slagelse-Ringsted Hospitals, Slagelse, Denmark; 11 Research Unit for Musculoskeletal Function and Physiotherapy, Department of Sports Science and Clinical Biomechanics, University of Southern Denmark, Odense, Denmark; 12 Department of Physiotherapy and Occupational Therapy, Zealand University Hospital, Roskilde, Denmark; 13 Institute of Psychology, University of Copenhagen, Copenhagen, Denmark

**Keywords:** Non-small-cell lung cancer, Health services, Prognosis, Quality of life, Signs and symptoms

## Abstract

We developed the Navigate intervention to improve survival among vulnerable lung cancer patients. In this intervention-only study, we examined feasibility in terms of *recruitment*, *retention*, *attendance*, *adherence,* and *acceptability* to specify adjustments to study procedures and intervention components prior to a randomized trial. The Navigate intervention includes nurse navigation, patient-reported outcomes, and physical exercise. Patients ≥ 18 years old, diagnosed with non-small cell lung cancer at any stage, with performance status ≤ 2, eligible for cancer treatment and vulnerable according to a screening instrument were included. The recruitment goal of eligible patients was 40% while the retention goal was 85%. The predefined cut-offs for sufficient attendance and adherence were ≥ 75%. Acceptability was evaluated by semi-structured interviews with participants, nurse navigators, and physiotherapists. Seventeen (56%) out of 30 screened patients were considered vulnerable and eligible for the study, 14 (82%) accepted participation, and 3 (21%) were subsequently excluded due to ineligibility, leaving 11 patients. Four patients dropped out (36%) and four patients died (36%) during follow-up and 3 (27%) were retained. All 11 patients participated in nurse sessions (mean 16, range 1–36) with 88% attendance and dialogue tools being applied in 68% of sessions. Ninety-one percent of patients responded to PROs (mean of 9 PROs, range 1–24) with 76% of the PRO questionnaires used (attendance) and 100% adherence (completion of all questions in PRO questionnaires), and 55% participated in exercise sessions with 58% attendance and 85% adherence. We identified important barriers primarily related to transportation, but overall acceptability was high. The Navigate intervention was feasible with high participation, acceptability and satisfactory adherence. Retention and exercise attendance were low, which resulted in adjustments.

**Trial registration:** The feasibility study was initiated prior to the multicenter randomized controlled trial registered by ClinicalTrials.gov (number: NCT05053997; date 23/09/2021).

## Introduction

Social disparities exist both in lung cancer incidence, survival^1–3^ and receipt of first-line guideline-recommended treatment^2,4,5^. Adverse health behavior such as smoking, high alcohol consumption and limited physical activity, poor physical condition and limited psychosocial resources^6,7^ may influence both treatment decisions and inhibit the patient’s ability to adhere to treatment. Other contributing factors may include inadequate time in consultations with patients and knowledge of how to communicate with vulnerable patients among healthcare professionals^8,9^ and lack of resources in the healthcare system^10^. The social gradient in both patient-near and system-related factors may result in lower levels of emotional support, participatory dialogue, involvement in treatment decisions and treatment information for patients with fewer resources^8,9,11,12^. No previous interventions have aimed to improve treatment and rehabilitation adherence^13,14^ among lung cancer patients at risk of non-adherence to treatment and follow-up. We developed the Navigate intervention^15^ including nurse navigation^16–18^, use of patient-reported outcomes (PROs)^19–21^ and physical exercise^22,23^ to improve survival for lung cancer patients who are vulnerable in terms of social, behavioral and disease factors.

To ensure high-quality trials, it is of key importance to assess the feasibility of interventions especially when the patient group has not previously been included in clinical trials. While measures of adherence may inform the delivered dose of interventions and the understanding of trial results, acceptability from the perspective of both healthcare professionals and patients may help identify intervention characteristics crucial to adherence^24,25^. Few previous studies have described feasibility aspects of nurse navigation^26^, use of PROs^20,27,28^ and physical exercise^23,29,30^ among lung cancer patients, and none systematically included patients who were socially, behaviourally, and physically vulnerable. Only one previous randomized controlled trial (RCT) tested a tailored supportive care intervention among 108 inoperable lung cancer patients and reported high adherence (87%)^26^. However, the intervention was brief with only two consultations. Two feasibility studies of web-based PRO monitoring in unselected patients with lung cancer found high adherence rates (93% and 94%)^27,28^ and one of these studies reported high acceptability for both patients and healthcare professionals^28^. Previous feasibility studies of 6–8 weeks exercise interventions among lung cancer patients achieved between 44 and 87% attendance rates for supervised exercise sessions^31–34^, while the attendance was low for home-based exercise programs^31,32^. Two of these studies evaluated aspects of acceptability from the patients’ perspective showing high acceptability of hospital-based exercise programs^32,33^ and low acceptability for a walking and relaxation program in home-based settings with key barriers being lack of self-discipline and doubts concerning the effects^32^.

In preparation for the ongoing multicenter RCT, the current study aimed to determine the feasibility and acceptability of the Navigate intervention in order to identify needed adjustments of the intervention components and study procedures including questionnaires and evaluate the expected goals for recruitment, retention, attendance and adherence.

## Methods

### Design and setting

The study was conducted as an one-armed, intervention-only feasibility study of the Navigate intervention targeting vulnerable lung cancer patients. Recruitment took place between October 2021 and January 2022 at the Department of Clinical Oncology and Palliative Care, Zealand University Hospital, Roskilde and patients were followed until January 2023. The Consolidated Standards of Reporting Trials (CONSORT) statement for feasibility and pilot studies was used^35^ (Supplementary Table 1). The Ethics Committee, Region Zealand (SJ-884/EMN-2020-37380) and the Data Protection Agency in Region Zealand (REG-080-2021) approved the study, participants provided written informed consent and all methods were performed in accordance with relevant guidelines and regulations.

### Eligibility criteria

All patients diagnosed with non-small cell lung cancer at any stage, with an Eastern Cooperative Oncology Group performance score up to two^36^, above 18 years, eligible for cancer treatment and screened vulnerable according to a screening instrument within 1 week of diagnosis were eligible for the study. The screening instrument was developed through literature review and with feedback from clinical experts and lung cancer patients^15^ to identify patients, who were vulnerable in terms of social, behavioral and health (both cancer and non-cancer) factors and at risk for not adhering to treatment guidelines and follow-up. Patients were screened vulnerable if they met three or more of the following nine criteria: (1) stage IIIB-IV (diagnosed with large tumours that have spread to nearby lymph nodes or other areas of the body), (2) comorbidity (somatic disease, e.g. heart disease or mental disease, e.g. depression) with impact on treatment or comorbidity resulting in hospitalization within last three years, (3) above 80 years, (4) performance status of two (and not above two as this is an exclusion criterion), as well as self-reported measures of (5) difficulties in activities of daily living, (6) low social support from social network, (7) low health literacy, (8) transportation-related barriers for treatment or (9) alcohol abuse. Patients were excluded if they were not able to read and understand Danish or had a severe untreated psychiatric disorder or cognitive problems preventing them from giving informed consent.

#### Intervention program

The development and content of the Navigate intervention have previously been described in detail^15^. Briefly, the one-year intervention included three components: nurse navigation based on Motivational Interviewing (MI)^37^, use of PROs and physical exercise.

The manualized nurse navigation sessions were structured with the aim to (1) optimize symptom management, (2) increase treatment initiation and adherence and (3) support patients in health behavior changes and psychosocial needs. Face-to-face or telephone (by patient’s preference) nurse sessions were offered by the following intervals: weekly during the exercise program, bi-weekly while still receiving treatment and monthly after end of treatment.

The aim of systematically collecting PROs was to initiate timely medical treatment or self-management strategies to lower the symptom burden, increase health-related quality of life and treatment adherence. PRO screening for symptoms was monitored bi-weekly throughout the one-year intervention either electronically or through telephone interviews with the nurse-navigator, as per patients’ preference. Patients reported on twelve physical symptoms adapted from the European Organization for Research and Treatment of Cancer (EORTC)^21,38^. The nurse-navigator followed pre-defined recommended actions according to each elevated symptom, e.g., appointment with an oncologist or self-management strategies.

The manualized exercise program aimed to prevent a decline in physical function and enhance the level of physical activity to improve eligibility for cancer treatment and treatment adherence. The program included 24 exercise sessions (twice weekly) supervised by physiotherapists over the first three months of the intervention targeting cardiorespiratory fitness (15 min of aerobic exercise such as walking or cycling with intensity level 14–15 on the BORG scale) as well as muscle strength and endurance including 25 min of pull-to-chest, sit-to-stand, shoulder press and abdominal crunch with three sets of 15 repetitions in a sitting position with elastic bands in different strengths. Tailoring was possible through manualized criteria for the progression or regression of the intensity level. The first eight exercise sessions were supervised at the hospital to ensure participants could perform the program at home if needed. Any home-based sessions were supported by an online video-based exercise guide available at the study’s webpage and the physiotherapists guided patients by telephone if they needed help with the web browser or any of the exercises. Patients were instructed to fill out an exercise diary to support and document their training at home and to monitor any minor or severe adverse advents occurring in connection with the exercises: ‘Did you feel any discomfort or changed the exercise?’. Physiotherapists were instructed to follow up on the patient’s diary each week to document the exercises or any adverse events occurring in connection with the exercise program in Redcap.

### Data collecting by questionnaires and physical testing

Patients filled in questionnaires (demographics (baseline only), health-related quality of life (The European Organisation for Research and Treatment of Cancer Quality-of-life Questionnaire Core 30, Quality-of-life Questionnaire Lung Cancer 13 and European Quality of life Questionnaire-5 Dimensions-5 Levels), health behaviour (alcohol, smoking and physical activity measured by single-item questions), self-activation/self-efficacy (single items from Patient Activation Measure and Health Education Impact Questionnaire) and rehabilitation services (measured by a single question) at baseline and after 3, 6 and 12 months either electronically, on paper or via telephone as per the patients’ preference. Students in medical sciences were trained to perform the questionnaire interview by telephone and probe to support patients through the questions.

To assess and motivate physical activity, patients were monitored with pedometers over 7 days handed out at the baseline physical tests including a prepaid envelope to return the pedometer after 7 days.

Physical fitness was evaluated at baseline, at the end of the exercise program and at 12 months follow-up. Cardiorespiratory fitness was assessed using the Ekblom-Bak test^39^, endurance and walking ability were assessed using the six-minute walk test (6MWT)^40^, muscular strength using the 30-s chair stand test^41^ as well as using a handgrip dynamometer (Jamar) test^42^. The Ekblom-Bak test was conducted on a cycle ergometer where patients were instructed to pedal at a low work rate of 0.5 kiloponds with a pedal frequency of 60 revolutions per minute (RPM) during the first 4 min followed by 4 min on a higher individually chosen work rate corresponding to 14 on the BORG scale^43^. The 6MWT was performed in a 20-m undisturbed hallway while the 30-s chair stand test was performed with the patient seated on a 44–47 cm highchair having a straight back, feet approximately shoulder-width apart and placed on the floor with arms crossed over the chest. The patients were encouraged to complete as many full stands as possible within a 30-s time limit. The handgrip test was performed with three measurements on each hand using the best test result from each hand. The physiotherapists were trained to perform the testing procedure.

#### Feasibility evaluation

Feasibility was examined in terms of recruitment, retention, attendance and adherence to evaluate the expected goals for the ongoing multicenter RCT, while acceptance was evaluated among patients, nurses and physiotherapists to examine whether study procedures or intervention components required any adjustments.

### Recruitment and retention

Based on previous trials among lung cancer patients^23,26^, the expected recruitment goal was 40% while the retention goal at 12 months follow-up was 85%. We evaluated the recruitment goal as the number of patients agreeing to participate in the Navigate pilot study out of all eligible and approached patients. The retention goal was evaluated as the number of patients alive and remaining in the study at 12 months follow-up out of the number of patients recruited into the study.

### Attendance and adherence

The predefined cut-offs for sufficient attendance and adherence were ≥ 75%. We calculated attendance for each intervention component according to the individual patient’s follow-up time, e.g., the number of exercise sessions divided by the number of possible sessions until dropout. Adherence to the nurse manual was calculated as the number of nurse sessions using manual dialogue tools out of the total number of nurse sessions. We calculated adherence to PRO questionnaires as the average number of questions completed by the participants out of the total number of 14 questions in the PRO questionnaires. Finally, adherence to the exercise program was calculated as the average minutes of aerobic exercises out of the total of 15 min of exercise and average repetitions per set of strength exercises out of the total of 15 repetitions, as well as the average intensity out of the prescribed amount (15 on the BORG scale and 6 on the OMNI scale).

#### Acceptability

We evaluated acceptability by semi-structured face-to-face interviews with participants after 3 months and with nurse navigators and physiotherapists after 12 months applying seven constructs of the Theoretical Framework of Acceptability (TFA)^25^. Interviews were transcribed verbatim and data extraction and condensation were done including familiarization with the interview, using deductive coding and summarizing data according to the seven constructs of TFA^25,44^. Moreover, patient-reported acceptability on a Likert scale (single items) was obtained after 12 months from questionnaires. We defined high satisfaction with the intervention as a minimum of 75% of patients rating each intervention component as either very relevant or relevant and highly beneficial for them.

### Feasibility of study procedures

We evaluated the feasibility of self-reported questionnaires (electronic, paper or telephone) at baseline and after 3, 6 and 12 months through patient interviews. The feasibility of the physical tests was evaluated at baseline in terms of acceptance and adherence by feedback from the physiotherapists.

### Statistics

As this is a feasibility study, a sample size calculation was not performed. We had originally planned to include 20 participants, but this was revised to 15 participants as this was expected to be sufficient to reach information saturation regarding feasibility aspects for the ongoing RCT^45^. Descriptive statistics was performed to estimate the frequencies, means and SD of the baseline patient, clinical and treatment characteristics. We estimated the rates of recruitment, retention, attendance and adherence in numbers and percentages.

### Ethics statement

Ethics Committee, Region Zealand (SJ-884/EMN-2020-37380) and the Data Protection Agency in Region Zealand (REG-080-2021) approved the feasibility study. Participants provided written informed consent.

## Results

**Figure 1:**
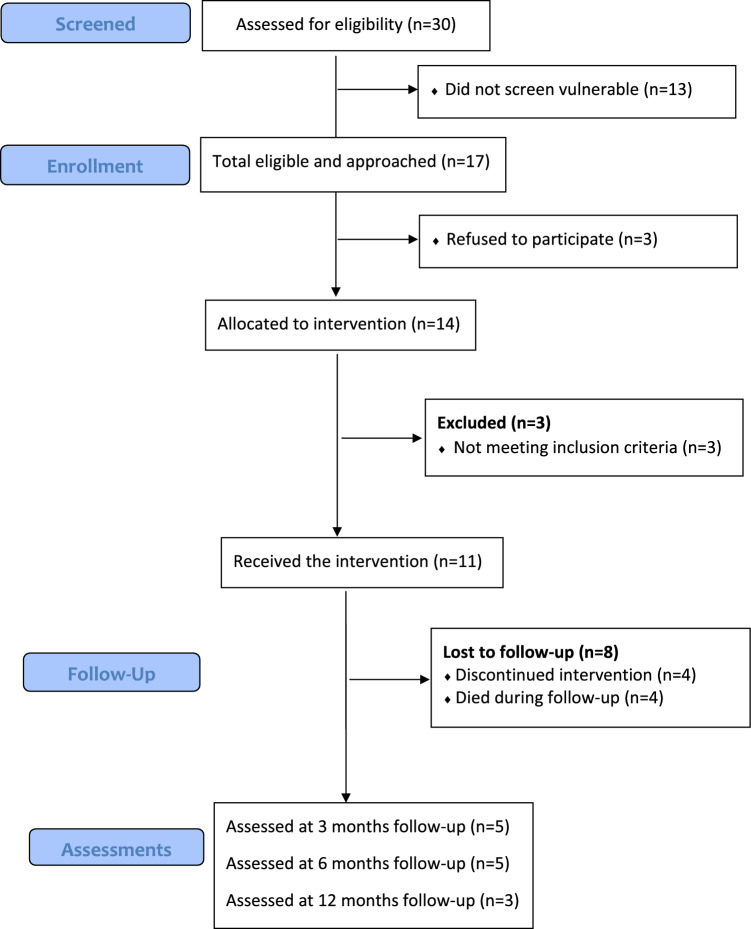
Participant flow.

From October 2021 to January 2022, we screened 30 lung cancer patients for vulnerability with 56% (N = 17) considered vulnerable and eligible for the study. Out of the 17 eligible patients, 14 patients (82%) agreed to participate (Fig. 1) with the last follow-up in January 2023. Three patients were subsequently excluded due to ineligibility (too cognitively impaired to participate or rapid disease progression resulting in a performance status of 3) and did not receive the intervention leaving 11 patients in total (7 males and 4 females; mean age 73 years, SD 6). Baseline characteristics for the 11 patients are shown in Table 1. Most patients were screened as vulnerable due to an advanced disease stage (Table 2). Moreover, approximately one-third reported having no help with practicalities at home and difficulties filling in forms or reaching the hospital due to lack of transportation or long travel distance (Table 2). Four patients dropped out (36%) and four patients died (36%) during follow-up and retention at 12 months was 27% (3 out of 11 patients). The primary reasons for dropout were no energy or being too sick to participate. In general, the patients were satisfied with the intervention describing positive attitudes regarding each intervention component (Table 3) and considered nurse sessions and the exercise program as relevant (100% and 68%, respectively) and the intervention as highly beneficial for them (68%).

**Table 1:** Baseline characteristics among 11 participants in the Navigate feasibility study.

Patient characteristics	Number	%
Age, mean SD	73	6
Gender		
Male	7	64
Female	4	36
Marital status		
Married or cohabiting	6	55
Single	5	45
Educational attainment		
Short	2	18
Medium	6	55
Higher	3	27
Employment status		
Employed	0	0
Unemployed, long-term sickness leave or disability pensioner	2	18
Old age pensioner	9	72
Comorbidity		
Yes	9	82
No	2	18
Performance status		
0	1	10
1	5	45
2	5	45
Stage		
I–IIIA	2	18
IIIB–IV	9	81
Treatment type		
Curative	1	9
Palliative	9	82
Missing	1	9
Smoking status		
Current	4	36
Previous	7	64
Never	0	0
Alcohol consumption of five units		
Daily	1	9
Weekly	1	9
Monthly or fewer	7	64
No alcohol	2	18
Alcohol recommendations WHO		
Below recommend	7	64
Above recommend	4	36
Physical activity, vigorous		
None	8	73
Up to 1 h	2	18
Between 1–2 h	1	9
More than 2 h	0	0
Physical activity, moderate		
None	7	64
Up to 1 h	1	9
Between 1–2 h	3	27
More than 2 h	0	0
Sedentary behavior		
Almost all-day	4	36
10–15 h	3	27
4–9 h	4	36
Never or up to 3 h		
I have a very good idea of how to manage my health problems		
Strongly disagree	0	0
Disagree	3	27
Agree	5	45
Strongly agree	3	27
I am confident that I can take actions that will help prevent or minimize some symptoms or problems associated with my health condition		
Disagree strongly	0	0
Disagree	3	27
Agree	7	64
Agree strongly	1	9
I have been able to maintain the lifestyle changes for my health condition that I have made		
Disagree strongly	1	9
Disagree	1	9
Agree	8	73
Agree strongly	1	9
EORTC-30 health-related quality of life (high score represents a high/healthy level of functioning)		
Global health status, (mean, SD)	55.3	24.5
Physical functioning, (mean, SD)	56.9	18.9
Role functioning, (mean, SD)	40.9	31.0
Emotional functioning, (mean, SD)	68.1	22.9
Cognitive functioning, (mean, SD)	86.3	14.5
Social functioning, (mean, SD)	83.3	23.5
Lung cancer-specific symptom scale (high score represents a high symptom burden)		
Fatigue, (mean, SD)	56.5	16.8
Nausea and vomiting, (mean, SD)	7.5	20.2
Pain, (mean, SD)	28.7	27.9
Dyspnoea, (mean, SD)	63.6	31.4
Insomnia, (mean, SD)	42.4	33.6
Appetite loss, (mean, SD)	21.2	34.2
Constipation, (mean, SD)	21.2	30.8
Diarrhoea, (mean, SD)	12.1	22.4
Financial difficulties, (mean, SD)	3.0	10.0
Physical tests		
Walk test, (mean, SD)	277.4	115.1
Sit-to-stand test, (mean, SD)	8.6	3
Grip strength right, (mean, SD)	27.5	7.8
Grip strength left, (mean, SD)	26	9.2

**Table 2:** Vulnerability characteristics at lung cancer diagnosis among 11 patients.

Vulnerability criteria	Number	%
Patient-reported criteria		
Activities of daily living		
Difficulties with eating and personal hygiene	3	27
Difficulties with taking a short walk outdoors	6	55
Difficulties climbing several stairs	10	91
Social support from social network		
No transportation assistance to the hospital	6	55
No help with practicalities at home	4	36
No emotional support	0	0
Transportation-related barriers		
Difficulties in reaching the hospital due to lack of transportation	4	36
Difficulties in reaching the hospital due to long distance	3	27
Difficulties in reaching the hospital due to limited energy	2	18
Alcohol abuse		
Drinking more than 7 units during a normal day	2	18
Most often drink more than 6 units daily	1	9
Health literacy		
Difficulties in understanding healthcare information	2	18
Difficulties in understanding instructions from healthcare professionals	1	9
Difficulties in filling in forms	3	27
Clinical criteria from the medical journal		
Stage IIIB–IV	11	100
Comorbidities	11	100
Age > 80 years	2	18
Performance status 2	3	27

**Table 3:** Evaluation of acceptability of the intervention from the perspective of patients, nurses and physiotherapists.

Theoretical framework of acceptability constructs	Patients’ perspective	Nurses’ perspective	Physiotherapists’ perspective
**Affective attitude** How an individual feels about the intervention	*The PRO questionnaire was acceptable and could identify problems that the nurse could help with* *Continuity with the same nurse was important to give patients a sense of comfort and trust. It was very rewarding for patients that they could influence the direction of the dialogue. Patients were relieved that the nurse had the time needed to talk and did not find it uncomfortable to be asked about smoking and alcohol, but rather it would have been untrustworthy if it was unmentioned* *Patients seemed very positive about the physical exercise program, especially the simplicity of the program. Initially, patients seemed reluctant, but after being informed about the importance of being physically active, most patients did understand the purpose*	*Nurses believed that the sessions gave the possibility to accomplish so much with the patients. Trust was created by having time to listen to patients’ life stories* *It was essential for nurses to feel motivated by communication and engaging with patients and to consider these aspects as essential professional skills to support patients in their needs and towards stages of change*	*Overall, physiotherapists were positive about the exercise program. The volume of time, intensity and type were well-suited for the patient population. In general, all included patients were regarded as ready for exercise without no special precautions*
**Burden** The perceived amount of effort that is required to participate in or deliver the intervention	*It was a challenge for patients to plan and perform the exercise program at home and to go for walks when it was dark outside, and the weather was bad*	*Nurses found it difficult to manage both patients outside the project and Navigate patients with the process of going from one setup to another being quite demanding* *Nurses had difficulties, especially in the beginning, figuring out exactly how to use the extensive intervention manual*	*The vigorous physical exercise presented symptoms similar to the disease-specific symptoms, so it was important to provide patients with the reassurance of safety* *Some physical performance tests were heavily burdensome. No patients were able to perform the Ekblom-Bak test. Therefore, the test was left out. Further, pedometers would provide objective measures of daily physical activity, but patients were unable to understand instructions due to their complexity* *A burdensome aspect of the physical exercise program was the transportation to the exercise facility. Most patients exercised in home-based settings due to transportation barriers*
**Ethics** The extent to which the intervention has a good fit with an individual’s value system		*Nurses checked with patients who started to cry whether it was too intense for them and if they wanted to stop to be sure that their boundaries were not crossed*	*Physiotherapists reported ethical concerns when asking patients to exercise vigorously, in the last period of their lives*
**Intervention coherence** The extent to which the participant or health professional understands the intervention and how it works	*Important to continuously revisit the project to make patients understand the purpose and content*	*The meetings with the project group during the study helped nurses to understand the manual in greater detail and to find possible solutions to problems*	*The instruction videos and exercise manual were not used systematically. Most importantly, to ensure an optimal understanding of the exercise program there was a strong need for a supervised exercise session, where physical instruction could be performed*
**Opportunity costs** The extent to which benefits, profits or values must be given up to engage in the intervention		*Important that colleagues and management support the project*	*Some patients declined to participate in the exercise program because they did not want to use all their remaining time on transportation to the exercise facility*
**Perceived effectiveness** The extent to which the intervention is perceived as likely to achieve its purpose	*The PRO questionnaire gave patients more insight into the disease and its symptoms. The nurse’s guidance enabled patients to better cope with their symptoms* *Patients experienced trust and acceptance with the nurse, which made them share difficult things in their life* *Regular follow-up was very important to maintain the desired change* *One patient stated that she did not know that exercise was so important for her treatment. She believed that the chemotherapy would fix it all* *One patient stated that if he had not continued with the exercise program, he would have lost his ability to walk and other physical functions*	*Nurses believed they had made a world of difference, especially for the most vulnerable patients* *One of the patients was unable to react to acute symptoms and the nurse could sense on the phone something was wrong and had the patient admitted to the emergency room* *Patients who never would have done exercise said that they could feel a physical improvement and that it motivated them to continue. But they nevertheless struggled with getting the exercises done at home, so it helped them enormously that the nurse called and followed up*	*Physiotherapists believed that the physical exercise program was beneficial. Either the program could enhance physical capabilities or at least serve as prevention of deterioration. Special emphasis was put on aerobic exercise. The strength training was not seen as “hard enough” for some of the patients* *The predominant home-based exercise settings might provide insufficient physiological stimuli, as supervision and monitoring of intensity are not possible*
**Self-efficacy** The participant’s or health professional’s confidence that they can perform the behavior (s) required to participate in the intervention	*It gave the patients a sense of confidence knowing that the same nurse would call and ask about their needs. The fact that the nurse asked about smoking made some patients smoke fewer cigarettes* *The possibility of doing exercises at home helped the patients tremendously* *The nurse asked the patients about exercises at home in a way that motivated the patients and gave them the confidence to continue.’*	*Supervision meetings with an expert in Motivational Interviewing were of key importance to support nurses in their new role as nurse navigators and enabled them to use the manual to a greater extent and develop their Motivational Interviewing skills*	*The bodily experience of successfully performing physical exercise was highly important. The “aha moment” of being able to walk for 6 min or get up from a chair numerous times, was linked to an enhancement in patients’ beliefs in themselves* *Opposingly, when their illness progressed, the exercises served as a visual representation of deterioration, which potentially could decrease self-efficacy*

### Nurse sessions

All 11 patients participated in nurse sessions (mean 16 sessions, range 1–36) with an attendance rate of 88% (Table 4). Most sessions were by telephone (87%) primarily due to transportation barriers. Nurse navigators used dialogue tools from the manual in 68% of the nurse sessions (Table 4). The most frequently used dialogue tool was asking the patients what is most important for them and setting an agenda as well as a dialogue tool to support patients in talking about difficult issues related to their disease. Nurse sessions lasted approximately 1 h (range 15–75 min). In total, nurses made 24 referrals, primarily to oncologists, social workers or psychologists. Patients experienced trust and acceptance from the nurse, which made them share difficult things in their life (Table 3). The nurses also expressed positive attitudes towards the intervention, e.g., that their support was highly beneficial, especially among the most vulnerable patients, but also challenges related to the work conditions, professional skills and the extensive intervention manual (Table 3). Supervision meetings were essential to support nurses in their new role as nurse navigators and enabled them to develop their MI skills.

**Table 4:** Attendance and adherence to each intervention component.

Intervention component	Attendance			
	Attended	Per manual	Range	Rate (%)
Nurse sessions (number)	171	195	1–36	88
PRO questionnaires (number)	106	139	1–24	76
Exercise sessions (number)	70	120	4–24	58

	Adherence			
	Adherence	Per manual	Range	Rate (%)
Dialogue tools used in nurse sessions (number, nurse sessions)	116	171	N.A.	68
PRO questionnaires (number)	106	106	N.A.	100
Aerobic exercises (mean, minutes)	12.75	15	11.9–15	85
Strength exercises (mean, repetitions per set)	12.75	15	6.5–15	85
Intensity BORG scale 6–20 (mean)	14.13	14–15	13–15.8	100
Intensity OMNI scale 0–10 (mean)	6	5–7	4.2–8.3	100

*N.A.* not applicable.

### PRO questionnaires

Ninety-one percent of patients (10 out of 11 patients) responded to the PRO questionnaires (mean of 9 PROs, range 1–24) with 106 out of 139 possible PRO questionnaires used until dropout due to death or withdrawal (76% attendance) (Table 4). All PRO questions in the 106 questionnaires were completed (100% adherence) by telephone and dyspnoea and fatigue were the most reported symptoms. Responding to the PRO questionnaire provided patients with knowledge on their disease, the nurse’s guidance enabled patients to better cope with their symptoms, and patients only had difficulties in relating to one question concerning whether they were depressed (Table 3). Nurses made six referrals to an oncologist and three acute referrals as a result of reported symptoms in PROs.

### Exercise program

Fifty-five percent of patients (6 out of 11 patients) participated in the exercise program (mean 5 sessions, range 4–24) and five did not participate due to early dropout (N = 3) or treatment complications resulting in severely impaired physical function (N = 2). The six patients attended 70 exercise sessions (58%) out of 120 maximum possible exercise sessions until drop-out due to death or withdrawal (Table 4). As patients had transportation barriers, the majority performed the exercise sessions at home (78%). The primary reasons for non-attendance were treatment-related fatigue or other burdensome symptoms. Adherence to the prescribed aerobic and strength exercises and intensity level among the six patients who participated in the exercise program was good (85–100%) (Table 4). The primary reasons for non-adherence were exhaustion. No severe adverse events during exercise sessions were reported, but minor issues related to muscle soreness (n = 4 patients) and lactic acid in legs (n = 1 patient) did occur. Overall, physiotherapists and patients reported high acceptability of the exercise program and found the volume of time, intensity and type of exercise well-suited. The bodily experience of successfully performing physical exercise was linked to an enhancement in the patient’s self-efficacy (Table 3).

### Feasibility of study procedures

Patients found several physical assessments and study procedures challenging. Firstly, the use of pedometers to assess and motivate physical activity over 7 days was too demanding, as patients either forgot to use them or did not return them. Secondly, none of the patients were able to complete the Ekblom-Bak test at baseline, primarily due to poor physical condition and severe dyspnoea. Finally, most patients were not able to attend the baseline physical tests primarily due to transportation barriers prolonging the inclusion of patients up to 1.5 months from the day of recruitment. Conversely, the possibility for patients to select a questionnaire format (electronic, paper version, or telephone-based) that matched their preference reduced the patient burden.

## Discussion

This feasibility study of the first intervention targeting vulnerable patients with lung cancer illustrated a high participation rate, adherence, acceptability, and thus relevance of the intervention. However, several intervention components and study procedures required tailoring. Moreover, since retention was low (27%) and only approximately half of the participants attended the exercise program these goals needed adjustment for the ongoing multicentre RCT.

The high inclusion rate of 82% was expected due to the intervention-only study design. However, 21% were subsequently judged ineligible primarily because of rapid disease progression to a terminal phase, which resulted in a discussion of more careful procedures for evaluating eligibility. Moreover, as also recognised in previous studies once recruited into the study, retaining this vulnerable patient group in the intervention was a challenge as they had limited mental and physical resources, e.g. due to treatment complication-related hospital admissions^46^. Our ongoing RCT should expect lower recruitment rates due to the randomized study design, exclusions due to ineligibility and low retention rates for this vulnerable patient group. It is important to note that the proportion of low retention due to death (36%) will not limit analyses of the primary outcome of survival, but the analyses of the secondary outcomes. To evaluate potential consequences of low recruitment and retention rates for the statistical power and to potentially adapt sample size, we plan to conduct interim analyses 2 years after start of inclusion.

### Nurse sessions

Patients had high nurse session attendance (88%) and due to transportation barriers, in-person meetings were scheduled adjacent to planned hospital appointments and the manual requirement that the first nurse session should be in-person was dropped. The frequency of nurse sessions was quite extensive compared with a previous RCT among 108 inoperable lung cancer patients testing a tailored supportive care intervention with only two consultations^26^. Nevertheless, the attendance rate in our study (88%) was comparable with the brief intervention (87%)^26^ indicating that the nurse navigator support was acceptable for both patients and nurses. However, nurses also identified burdens related to the work conditions, professional skills and the extensive intervention manual. Organizational support at each participating site will be enhanced through continuous engagement of leaders during meetings and project presentations. Moreover, supervision concerning the use of MI techniques and support in manual use and study procedures will be continued throughout the RCT study period.

### PRO questionnaires

In agreement with our study, high adherence rates (93% and 94%) have been reported previously in studies of web-based PRO monitoring among patients with lung cancer^27,28^ as well as high acceptability for both patients and health care professionals^28^. Overall, the PRO questionnaire gave patients more disease insight and the nurses’ guidance enabled patients to manage their symptoms. As some patients had difficulties in relating to one question concerning whether they were depressed, this was replaced by a question concerning distress.

### Exercise program

Consistent with our findings, two RCTs of exercise interventions similar in frequency and timing (twice weekly sessions for 8–12 weeks), but with higher intensity levels have reported low attendance rates of 44% and drop-out rates of 32%–37%^23,34^. To provide the highest flexibility also for future implementation, we will allow patients in the RCT to decline participation in the exercise intervention component without being excluded from the study, even though this may dilute the intervention effect.

Previous studies have shown that lung cancer patients can adhere to the prescribed volume of exercise^47,48^, which is in line with our findings. Therefore, the adherence goal of 75% for the RCT seems realistic for patients attending the exercise program. The high acceptability of the exercise program in our study corroborates previous findings perhaps due to experiencing the effects of exercise in combination with support from a healthcare professional has positive effects on lung cancer patients’ attitudes towards exercise^32,49–51^. In line with our findings, transportation to the exercise facility has previously been identified as the most burdensome aspect of participating in supervised physical exercise^51^. Therefore, we will reduce the requirement of at least eight supervised sessions at the hospital to a minimum of one supervised session. We will address previous findings of low attendance in home-based exercise programs^31,32^ by a weekly follow-up on exercise sessions by the nurse navigator to reinforce motivation and thus attendance. This method provided positive results for some patients (Table 4).

### Planned analyses in the RCT

In addition to intention-to-treat (ITT) analyses in the RCT, we will also perform per protocol analyses exploring the potential full effect of the intervention by including only participants who meet the definition for sufficient attendance and adherence. Sufficient attendance will be defined as *participating* in 75% of PRO questionnaires (20 out of 26) and exercise sessions (18 out of 24) according to the manual. As nurse sessions are offered on a need basis, we are unable to define a sufficient proportion of sessions. Sufficient adherence will be defined as *completing* 75% of each intervention component according to the manual: (1) use of dialogue tools during nurse sessions, (2) all PRO questions in a minimum of 20 questionnaires and (3) aerobic and muscle strength exercises during minimum 18 sessions (aerobic exercise: time and intensity, muscle strength exercises: repetitions and intensity). This was the case for one-third of the participants in the feasibility study. In addition, since retention was low, we will perform an interim analysis in March 2024 to determine a sufficient sample size to evaluate survival in the RCT considering low retention rates.

### Feasibility of study procedures

Consistent with previous studies conducted on supportive care interventions for lung cancer patients^46^, we identified several research procedures that were challenging for the participants to comply with and that prompted changes for the RCT. Pedometers will not be used to assess and motivate physical activity and the Ekblom-Bak test will not be used to assess cardiorespiratory fitness. To reduce the inclusion duration, we will increase flexibility by allowing the baseline physical assessment to be performed after randomization to shorten the enrolment period. The impact of knowledge of group allocation on patients’ performance during the baseline physical test is expected to be minimal. To ensure that baseline odds of survival are the same for both groups in the RCT, patients will fill out questionnaires at baseline before randomization as soon as possible and within 1.5 months after being invited into the study^15^. With transportation as a key barrier, we will cover the costs related to transportation to the hospital.

The strength of this study was the exploration of both feasibility goals and acceptability of a complex intervention enabling the identification of key aspects to improve expected goals and study procedures for the ongoing RCT. The relatively low number of participating patients was a limitation of this study with key participation barriers being transportation, long distance to the hospital or limited mental or physical resources to comply with the requirements of participating in a research project. Moreover, since retention was low and mortality was high only three patients completed follow-up after 12 months.

## Conclusions

We explored the feasibility of the first intervention targeting vulnerable lung cancer patients. The results illustrate a high participation rate and high acceptability and thus the relevance of the intervention, but low retention, exercise attendance and barriers were identified leading to an adjustment of study procedures to meet the complex needs of the study population. The Navigate intervention is currently being evaluated in a multicenter RCT with recruitment start March 1st, 2022.

### Supplementary Information

Supplementary Information.

Supplementary Table 1.

## Data Availability

Due to the EU regulation, the General Data Policy Regulation, we cannot share data with external parties without prior consent of the participants. Collaboration projects will however be possible by contacting Co-PI Pernille Bidstrup: pernille@cancer.dk.
