# Supplementary material for: Nurse navigation, symptom monitoring and exercise in vulnerable patients with lung cancer: feasibility of the NAVIGATE intervention
Source: Sci Rep. 2023 Dec 20;13:22744. doi: 10.1038/s41598-023-50161-w (PMC10733288; doi:10.1038/s41598-023-50161-w)
Supplement: Supplementary file 1 — Supplementary Information. [file 41598_2023_50161_MOESM1_ESM.pdf]

## Study protocol NAVIGATE

Version 7. 05.10.2022

**Title: NAVIGATE – Improving survival in vulnerable lung cancer patients through individual support: a multicenter randomized controlled trial**

**Project Group**

Rikke Langballe, R.N. Ph.D.

Susanne Oksbjerg Dalton, MD, Ph.D. Professor

Mads Nordahl Svendsen, MD

Erik Jakobsen, MD, Ph.D. Associate clinical professor

Maria Iachina, statistician, Ph.D.

Randi Valbjørn Karlsen, R.N., MEd

Helle Gert, R.N, chief nurse manager

Camilla Kjærulff, R.N, ward nurse

Elizabeth Emilie Rosted, R.N, Ph.D.

Jeanette Ehlers, MD, chief physician

Pernille Bidstrup, psychologist, Ph.D. Associate professor

# Appendix 2

## Content

Introduction ........ 3
Background ........ 3
Lung cancer prognosis and treatment ........ 3
Factors influencing treatment outcome ........ 4
Interventions to improve lung cancer care ........ 5
Patient and nurse-navigation in cancer care ........ 6
Exercise programs for lung cancer patients ........ 7
Use of PRO in lung cancer care ........ 8
Aims ........ 9
Methods ........ 9
Design ........ 9
Inclusion procedure ........ 9
Inclusion criteria ........ 11
Vulnerability criteria ........ 11
Exclusion criteria ........ 12
Allocation to study groups ........ 12
NAVIGATE intervention program (intervention group) ........ 13
Nurse-navigation ........ 13
Exercise program ........ 14
PRO Screening ........ 15
Intervention manual ........ 16
Standard care (control group) ........ 16
Data collection ........ 17
Feasibility study ........ 19
Process evaluation ........ 19
Power considerations ........ 20
Statistical analyses ........ 20
Study period and budget ........ 21
Study period ........ 21
Budget ........ 23
Ethical considerations ........ 24
Dissemination of study results ........ 24
Risk mitigation plan ........ 25
Project organization ........ 25
Perspectives ........ 26
References ........ 27

# Introduction

NAVIGATE is a sub-study within the Danish Research Center for Equality in Cancer, COMPAS, committed to provide optimal cancer treatment to all Danish cancer patients irrespective of social status. We aim to develop and test an intervention to vulnerable patients with lung cancer, a disease which is strongly associated with low socioeconomic position (SEP) in both incidence and survival [1,2]. To our knowledge, this will be the first study to examine the effect of an individually tailored nurse navigation for vulnerable lung cancer patients including exercise training and systematic use of patient reported outcomes (PROs) to improve overall survival, treatment adherence, symptom burden and health related quality of life (HRQoL).

# Background

## *Lung cancer prognosis and treatment*

Lung cancer continues to be the most commonly diagnosed malignancy in men and women combined and the leading cause of cancer death worldwide [3]. Lung cancer symptoms are often vague and non-specific and the development of a tumor may consequently go unnoticed leading to patients being diagnosed in an advanced stage [4]. In Denmark, approximately 4800 new cases of lung cancer with a median age of 70 years were diagnosed in 2018 and more than half of these patients were in stage IIIB–IV at diagnosis (55%) [5]. The 5-year survival rate for patients diagnosed with advanced disease ranges from 12% for stage IIIB to 2% for stage IV, while patients diagnosed in an earlier stage have a 5-year survival rate of 60% (stage IA) [5]. Although the prognosis for lung cancer patients overall has improved during the last decade due to advances in medical treatment [6] with increasing overall five-year survival rates from 8% to beyond 15% [6], the prognosis is still poor.

A systematic review and meta-analysis from 2013 found that lung cancer patients with low SEP were less likely to receive surgery and chemotherapy compared with patients of high SEP regardless of stage or histology and health care system [7]. Additionally, a Danish nationwide cohort

study from 2013 found that non-small cell lung cancer (NSCLC) stage I-IIIa patients with short education, low income and who live alone were less likely to receive potential curative surgery [8]. In 2015, an extended nationwide cohort study of Danish lung cancer patients in all stages diagnosed during 2004–2010 reported that 46% of patients do not receive the recommended first-line treatment for the specific stage of disease and performance status, and that patients with high-stage cancer who lived alone were 17% less likely to receive standard treatment [9]. These findings were supported by recent population based studies from Sweden and Norway which in general report that patients with short education were at higher risk of not receiving first line treatment (curative or palliative) independent of stage resulting in lower survival rates among these patients compared with patients with higher education [10-12]. Differences in received treatment, stage and comorbidity may explain a large proportion of the social inequality in lung cancer prognosis both among early stage and advanced stage patients [9]. Low SEP may be an expression of multiple modifiable factors including adverse health behavior and limited psychological and social resources influencing the ability to react on high-risk symptoms and to navigate the health care system [13]. Thus, the underlying factors that drive both decisions of treatment and adherence to treatment and rehabilitation may be important intervention targets to improve outcomes for lung cancer patients with low SEP.

## *Factors influencing treatment outcome*

A large proportion of lung cancer patients have comorbidities such as cardiovascular disease or chronic obstructive pulmonary disease which may affect physical functioning and general health [14]. The vast majority of patients are former smokers [15] or current smokers [16,17], a large proportion of patients have a moderate or high alcohol consumption [16,17] and some patients are in poor nutritional status and physically inactive [18]. Thus, lung cancer patients are often in a poor physical condition (performance status) at diagnosis which plays an important role in treatment decisions and may reduce treatment effects [19,20] and impair survival [16]. Limited physical, psychological and material resources may limit the individuals’ perceived possibility for changing behavior on their own [18,21] and to stop smoking, change diet or initiate physical exercise might be extremely difficult [22,23]. Thus, it is important to address health behavior changes such as smoking cessation, healthy diet, alcohol moderation and increase physical activity to improve the general health condition

and eligibility for treatment. Potentially this may also reduce symptoms during treatment and improve treatment adherence and survival.

Engaging patients in treatment decisions while balancing considerations of potential benefits and harms may be particularly difficult for patients of low SEP [24], possibly because of limited health literacy. Physicians may be challenged in their communication with patients with low SEP because this patient group may have misconceptions about the disease potentially leading to under-treatment and poor adherence to treatment [25]. Therefore, supportive strategies should be adapted to communicate treatment information and encourage dialogue with patients with limited health literacy to improve treatment initiation and adherence.

Lung cancer patients are among the most burdened cancer patients in terms of debilitating physical (i.e. dyspnea, pain, fatigue, and severe weight loss) and psychological (anxiety and depression) symptoms [26,27]. Worsening of symptoms in lung cancer patients during treatment such as pain or dyspnea may be indicators of treatment complications that may require delay or reduction in treatment dosage [28]. Moreover, it has been shown that symptom burden may negatively impact treatment adherence: A Danish prospective study of 137 lung cancer patients found that patients with high physical-, role-, emotional- and social function and with low fatigue, pain, appetite loss, and dyspnea were more likely to complete first line oncological treatment [29]. Thus early identification of treatment related symptoms may be important to initiate treatment changes or improve management of the individual symptoms. In addition,  qualitative studies has suggested  that some lung cancer patients feel they struggle to get the support they need [30], possibly due to their limited health literacy and insufficient  knowledge of how to navigate the health care system to reach the relevant support. Patients with poor health literacy may need education in understanding their symptoms as well as knowledge and support in how to reach out for the care needed [31].

## Interventions to improve lung cancer care

Few studies have attempted to improve treatment adherence in vulnerable lung cancer patients. Studies mainly on other cancer groups have shown promising and relevant aspects in nurse-

navigation, physical training programs and use of PROs to address the challenges during cancer treatment.

*Patient and nurse-navigation in cancer care*

Studies that have pooled and analyzed data from patient navigation programs in the US demonstrated a moderate benefit on improving timely cancer care among patients diagnosed with breast, cervical, colorectal and prostate cancer [32,33]. One of these multicenter studies reported that patient navigation may eliminate delays in diagnostic resolution in disadvantaged populations and reduce disparities in cancer care [33].

Patient navigation is often performed by nurses who have experience with cancer patients who are familiar with the health care system to support patients throughout their cancer trajectory [34,35]. Nurse-navigation in lung cancer care has the potential to increase the proportion of patients receiving systematic therapy and shorten the time to delivery of treatment [34,35]. Two retrospective observational studies among lung cancer patients have compared diagnostic and treatment outcomes before and after the implementation of a nurse-navigation and found that it may improve timeliness in lung cancer care [36,37]. However, to our knowledge only one RCT from Australia has investigated the effect of a nurse-navigation program among lung cancer patients [38], while few RCTs from Canada and the US comprised patients with different cancer diagnoses including lung cancer [39-41]. None of these studies found significant differences between intervention and control groups on primary outcomes (patient experience, unmet needs, psychological morbidity, distress, and HRQol), but one study reported fewer problems related to psychosocial support, care coordination and obtaining information [40]. However, all these studies are small (N=14-113 patients with lung cancer) and with methodological limitations. Taken together, evidence is still needed for the effect of nurse-navigation on clinical outcomes among lung cancer patients.

We have in a pilot RCT among 50 breast cancer patients who had high psychological distress shown positive effects on distress, anxiety and depression of an intervention combining nurse-navigation with PROs and we are now testing it in a full scale RCT with 324 breast cancer patients

[42,43]. Nurse-navigation holds great potential for other patient groups with higher symptom burden and with fewer resources, but this remains to be established.

*Exercise programs for lung cancer patients*

A recent Cochrane review [44] including 6 RCTs (N=221 patients) found significant effects of >4 weeks exercise training at least once a week during treatment among advanced lung cancer patients on six-minute walk distance (6MWD) and HRQol, but not on specific physical or psychological symptoms or survival. Exercise training prior to lung cancer treatment, has only been examined in few trials and cohorts based on small sample sizes. A recent meta-analysis [45] (N=676 patients with NSCLC in 10 RCTS) found that hospital-based physical training (combined aerobic, resistance and inspiratory muscle training) lasting 1–4 weeks before treatment initiation with 1–3 sessions per week may improve functional capacity, dyspnoea and recovery including reducing risk of hospitalization and complications. In a more recent RCT from China by Liu and colleagues [46], patients with stage I-III NSCLC receiving thoracoscopic lobectomy (N=73) were randomized to control group versus home-based, two-week training program before treatment initiation consisting of aerobic, resistance and respiratory training, nutrition counseling and psychological support. This study found significant improvements in six-minute walk distance (6MWD) 30 days after surgery [46], but it had limitations including a small and fit study population who were eligible for curative surgical treatment representing only approximately 25% of lung cancer patients [47]. A somewhat larger RCT from Switzerland [48] including 151 patients diagnosed with stage I-IIIA NSCLC also reported significant increased cardio-respiratory fitness (VO2peak) and walking capacity (6MWD) among patients randomized to a hospital-based, high-intensity interval training program initiated 2-3 weeks before surgery versus usual care. Still, further physical training studies with larger sample sizes are needed to demonstrate survival benefits in lung cancer patients.

Training programs usually do not consider smoking and alcohol consumption. A recent meta-analyses across cancer types found that distance-based interventions were able to significantly increase smoking cessation, but not alcohol moderation [49]. Remarkably, a recent Cochrane review showed that no RCTs have tested smoking cessation interventions in lung cancer patients [15]. However, a recent systematic review and meta-analysis did show that individuals with low SEP

who participated in smoking cessation interventions were significantly more likely to quit smoking than were control participants [50]. Still, the authors recommended further development of existing interventions for smoking cessation targeting socioeconomically disadvantaged groups. Very Brief Advice interventions on smoking cessation following an 'ask, advise, assist' framework have been suggested to increase the success rate of attempts to quit smoking [51]. The method is a simple way to offer cessation support such as medication or referral for behavioral support and is currently being implemented in municipalities and hospitals in Denmark [52].

### *Use of PRO in lung cancer care*

In attempt to optimize quality of cancer care and increase patient involvement, PROs have been found to improve patient provider communication, symptom control, patient satisfaction and increased supportive care measures [53]. In lung cancer, PRO has been frequently used to evaluate HRQol in the treatment of lung cancer mainly using the European Organization for Research and Treatment of cancer Quality-of-life Questionnaire Core 30 (EORTC QLQ-C30) and the lung module (QLQ-LC13) [54]. Basch et al [28] tested the effect of a web-based weekly PRO, with automated alerts to prompt clinicians for worsening symptoms among 766 patients with metastatic cancer (breast, genitourinary, gynecologic, or lung). They found improvements in HRQoL, less frequent use of emergency rooms, longer treatment with chemotherapy, and more patients were alive at one year (75% compared with 69% in the control group). A similar RCT by Denis and colleagues [55] among advanced lung cancer patients (N=133) tested a web-based weekly PRO (incl. weight loss, appetite loss, weakness, pain, cough, breathlessness, depression, fever, face swelling, lump under skin, voice changing, and blood in sputum) compared with routine follow-up with regular CT scans. The study reported that the median overall survival in the experimental arm was significantly improved (19 months compared with 12 months in the control arm) [55] potentially due to early detection of adverse events and recurrence and better performance status at recurrence [55,56]. Together these studies indicate that close monitoring and adequate clinical reactions to key alert symptoms may have the potential to improve the length of and ensure appropriate treatment and quality of care [28], the efficiency of follow-up [55] and overall survival [28,55-57]. Inspired by the study by Denis et al [55], an ongoing RCT in Denmark (the ProWide study) is currently testing whether weekly monitoring of PROs adapted to a Danish setting can improve early detection of progressive disease

and improve survival for patients diagnosed with stage III-IV NSCLC or small cell lung cancer (still recruiting, unpublished).

## Aims

We aim to develop and test the effect of a new intervention targeting vulnerable lung cancer patients on overall survival as the primary outcome and on adherence to cancer treatment, symptom burden and HRQoL as secondary outcomes.

## Methods

### *Design*

We will in a multicenter two-armed open label randomized controlled trial test the effect of a nurse-navigation intervention including physical training and systematic use of PROs compared with standard care among vulnerable lung cancer patients. Participating departments in the multicenter setting include departments of oncology in Roskilde, Hillerød, Bornholm, Sønderborg and Gødstrup and departments of respiratory medicine in Vejle, Odense, Næstved, Aalborg, Silkeborg and Gødstrup.

### *Inclusion procedure*

During a 1.5-year period, consecutive newly diagnosed (< 1 week) lung cancer patients will be pre-screened for eligibility at first attendance at the nine participating departments where patients often are accompanied by relatives. Eligible patients are invited to participate by project nurses from clinical trial units at the nine participating departments. In case, there is no research trial unit at the department, project nurses will be recruited from the department. The project nurses will provide verbal and written information about the NAVIGATE study and hand out the following:

- Participant information (Appendix 3a for participants in the pilot study or Appendix 3b for participants in the RCT study)

- Leaflet published by the National Ethical Committee 'Before you make up your mind' ('Før du beslutter dig') (Appendix 4)
- Informed consent form (Appendix 5)
- Copy of the informed consent form
- Leaflet explaining data storage according to GDPR requirements (oplysningspligt) (Appendix 6)
- A prepaid envelope

The invitation procedure will take place in a consultation room allowing an undisturbed conversation. Patients with no companion will receive information about the right to have a companion and the project nurse will offer to schedule a new consultation in person as per patients' preference. After a consideration time of minimum 24 hours, a project nurse will contact the patients by phone, where they will be given the opportunity to learn more about the study and ask more questions.

If patients have no physical attendance at the department in the near future (within 3 months), the project nurse will send an information letter concerning participation in the NAVIGATE research project with the project nurse's contact information and asking the patient to contact the nurse. If the patient does not respond to the letter, the project nurse will send a new information letter with the following documents:

- Participant information (Appendix 3a for participants in the pilot study or Appendix 3b for participants in the RCT study)
- Leaflet published by the National Ethical Committee 'Before you make up your mind' ('Før du beslutter dig') (Appendix 4)
- Informed consent form (Appendix 5)
- Copy of the informed consent form
- Leaflet explaining data storage according to GDPR requirements (oplysningspligt) (Appendix 6)
- A prepaid envelope

# Appendix 2

If the patients responds by letter or phone, the nurse will contact the patient by phone to provide further information. If the patient does not respond following these two written information letters, the project nurse will contact the patient by phone to inform about the study.

If the patients are willing to participate in the study, the nurse will follow the guidelines for obtaining informed consent (Appendix 7). Patients who agree to participate in the study are asked to sign the informed consent and return it in the prepaid envelope. The project nurse will send a secured link to the patient's e-mail address with access to the baseline questionnaire (Appendix 8). If patients wishes to fill out the baseline questionnaire in paper, the project nurse will send it by mail with a pre-paid return envelope. When the project nurse has received the signed informed consent form and the baseline questionnaire, she will access a computer program and perform the randomization. The project nurse will inform patients immediately about the result of the randomization and consequences for further study participation and the nurse navigator about the included patients and group allocation for each patient. If randomized to the intervention group the nurse navigator will schedule the first meeting with the patients and inform the local physiotherapist about the included patients.

## *Inclusion criteria*

To be included in the study patients have to fulfill the following criteria:

- Age $\geq$ 18 years of age
- Diagnosed with NSCLC at all stages regardless of treatment intension at the participating departments
- Performance status $\leq$ 2
- Vulnerable according to pre-defined criteria

## *Vulnerability criteria*

# Appendix 2

All patients will as part of standard treatment at the participating departments be screened regarding vulnerability using both physician and patient reported data (cut-off for vulnerability >3 points) (Appendix 9).

Physician reported:

1) Stage IIIB-IV (+1)

2) Comorbidity (somatic or psychiatric) with impact on treatment or comorbidity resulting in hospitalization within last 3 years (+1)

3) Age $\geq$ 80 years (+1)

4) Performance status=2 (+1)

Patient reported:

1) Activities of daily living (ADL) (+1)

2) Social support (+1)

3) Health literacy (+1)

4) Barriers for treatment (+1)

5) Alcohol abuse (+1)

*Exclusion criteria*

Patients who fulfill the following criteria are excluded from the study:

- Severe untreated psychiatric disorder (e.g. psychosis) or cognitive problems (e.g. dementia) preventing informed consent
- not able to receive treatment
- Not able to read and understand Danish

*Allocation to study groups*

Participants will be randomized (1:1) to standard treatment plus the intervention (intervention group) or standard treatment and care (control group). The computer-based randomization will

ensure a balanced number of random assignments to the two groups in blocks of randomly varying sizes of six, eight or ten patients securing an equal distribution of patients in both groups.

## NAVIGATE intervention program (intervention group)

Based on guidelines on how to develop complex interventions to improve health and healthcare [58], review of the literature and feedback from clinical experts and patients we have developed the NAVIAGTE intervention program including nurse navigation, physical training and systematic use of PROs (Figure 1).

**Figure 1. Study flow and intervention components**

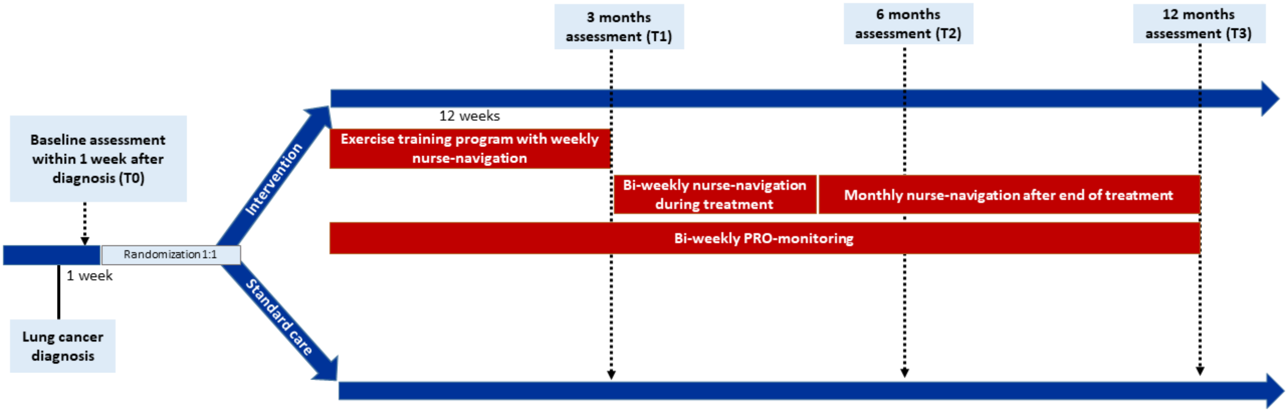


### *Nurse-navigation*

The aims of the nurse-navigation are:

- To identify patients with high-risk symptoms such as sudden worsening in pain or dyspnea who may be in need of changes in treatment or new medical management of symptoms.
- To motivate and support patients in making decisions about treatment, during treatment and in self-managing their symptoms in order to increase treatment initiation and adherence through frequent contact and follow-up.
- To motivate and support patients in health behavior changes such as increased physical activity, smoking cessation, healthy diet, alcohol moderation by initiating self-management

strategies and referral to existing rehabilitation services at the participating hospitals or at the local rehabilitation center.

The nurse-navigator will continue to follow-up on these aims throughout the 1-year intervention and tailor the intervention according to the individual patient. The nurse navigation will be based on the concept and techniques from motivational interviewing which has shown to be an effective strategy to enhance motivation and health behavior changes among patients with cancer [59]. The nurse navigator will guide patients in self-management strategies and sessions may include principles of emphatic listening and dialogue, psychoeducation, goal setting and agreement plans.

Nurse-navigation will be performed by 1-2 trained nurses at each department. Individual, manualized, face-to-face or telephone sessions are offered on a needs basis – tentatively bi-weekly during treatment and monthly after end of treatment. During the training program the nurse will contact the patients weekly to answer questions and evaluate compliance. All nurse-navigators will enroll in a 5 day training course prior to intervention initiation and will receive monthly supervision regarding patients and other study related issues.

### *Exercise program*

Patients will be offered a manualized 12 week training program. The aim of the training program is to improve the general health condition, eligibility for treatment and treatment adherence. The program will include components of aerobic, resistance and respiratory training and health behavior support and will be a combination of supervised training at the local rehabilitation center and home-based exercises (Appendix 10). The exercise program will be initiated as soon as possible after randomization – 4 weeks after randomization at the latest – and will consist of 24 exercise sessions with a duration of 45 minutes each performed 2 times per week. Intensity level of the aerobic exercises will be guided using the Borg's rating of perceived exertion scale 6-20. Exercise at Borg 11-14 will be considered as moderate-intensity and beyond 14 as vigorous-intensity. The exercise program will consist of the following exercises:

# Appendix 2

**Warm up** (5 minutes)

Can be completed on a stationary bike, as walking or other mode equivalent to this.

Intensity level: 11-14 on Borg scale.

**Aerobic exercise** (15 minutes)

Can be completed on a stationary bike, as walking or other mode equivalent to this.

Intensity level: 11-14 on Borg scale.

**Muscle strength/muscle endurance exercises** (25 minutes)

Standing dumbbell row (3 sets of 15 repetitions)

Sit to stand (3 sets of 15 repetitions)

Shoulder press (3 sets of 15 repetitions)

Abdominal crunch (3 sets of 15 repetitions)

At the baseline visit, the nurse navigator will perform assessments of aerobic, resistance and respiratory condition in collaboration with a physiotherapist as well as assessments of nutritional state, smoking and alcohol consumption. Patients with low weight will be recommended a high caloric diet. Patients at need will be provided with protein supplement for daily intake to achieve adequate protein intake, recommended as 1.5g/kg/day. The nurse navigator will support patients in smoking cessation by referral to smoking cessation programs at the local rehabilitation center and advice on nicotine replacement therapy using the Very Brief Advice method based on an 'ask, advise, assist' framework. Alcohol reduction support will include referral to alcohol addiction treatment programs at the local rehabilitation center using the Very Brief Advice method. The nurse-navigator will continue to motivate and support health behavior changes related to physical activity, smoking and alcohol throughout the 1-year intervention.

### *PRO Screening*

The aim of collecting PROs is to systematically monitor symptoms and initiate appropriate actions in terms of medical treatment or self-management strategies. PRO screening for symptoms will be collected through an electronic platform bi-weekly or alternatively through telephone interviews

with the nurse-navigator, as per patient preference, from diagnosis and up to 12 months. Patients will report on twelve physical symptoms adapted from the EORTC (self-rated health, weight loss, appetite loss, weakness, pain, cough, breathlessness, fever, face swelling, lump under skin, voice changing, blood in sputum) considered to be important to monitor during treatment with chemotherapy [55,60]. This will be supplemented with a measure of psychological distress using the Distress Thermometer [61] (Appendix 11). The results will be represented graphically to the nurse. A detailed algorithm will be developed which describes recommended actions by the nurse-navigator according to each elevated symptom, e.g. booking of appointment with oncologist or instructions regarding self-management of symptom.

### *Intervention manual*

A detailed manual will describe the content and the theoretical basis for the mechanisms used in the intervention as well as the response algorithm for PRO symptoms and the physical training component. This manual will work as a guiding tool for the nurse navigators and physiotherapists, and will document and ensure standardization of the intervention. Further, the manual will make it possible to evaluate to which extent the intervention was implemented as intended (implementation fidelity) [62] (further details can be found in the section 'Process evaluation').

## Standard care (control group)

Patients randomized to the control group will receive standard treatment and care consisting of a nurse and a physician, who sees the patient at treatment schedules and during follow-up, i.e. every 3 months for the first 2 years and subsequently annually up to 5 years. In some cases, shorter intervals are offered, e.g. if the patient has a poor performance status. At the first treatment schedule, the patient's physical, mental and social problems related to the lung cancer diagnosis and treatment are assessed as well as patient reported needs of support related to diet, smoking, alcohol and exercise using a standardized questionnaire. The patient's response is used to assess any side effects or rehabilitation needs as well as to refer to relevant rehabilitation services. The nurse will continue to assess potential side effects or psychological or social issues

during treatment and follow-up and if needed refer patients to a dietitian, social worker or priest. The treating physician can refer to a palliative care team if needed.

## Data collection

Before patients are invited to participate in the study, information about the inclusion and exclusion criteria (page 10-12) from the medical journal and the treating physician as well as data from the vulnerability screening (Appendix 9) will be disclosed to the project nurses performing the evaluation of eligibility for inclusion into the study.

Patients who provide informed consent will allow the use of data from self-reported questionnaires, medical journals and national registries in order to fulfill our research aim as well as for monitoring purposes (quality control and monitoring which we are obliged to perform):

- The intervention and the control group will be assessed at baseline within 1 month after diagnosis ($T_0$), and 3 months ($T_1$), 6 months ($T_2$) and 12 months ($T_3$) after diagnosis (see Figure 1). The questionnaires at each assessment will include the variables in Table 1. Considering that participating patients are vulnerable with limited resources we will proactively support patients in responding to questionnaires electronically, on paper or via telephone as per patient's preference allowing evaluation of the secondary outcomes as well as covariates and mechanisms.
- Information on vital status and clinical and treatment factors (Table 1) will be obtained from individual medical journals, the lung cancer clinical database and the Civil Registration System.
- In order to develop cost-utility analyses, use of health services including all outpatient visits to any healthcare clinic will be retrieved from the Danish National Patient Registry [63] and medical records while information on disability and productivity loss (sick leave, disability pension and retirement pension) will be obtained from the Integrated Database for Labor Market Research [64].

# Appendix 2

- We will use audio-recordings in order to assess implementation fidelity and for supervision; however, only among participants who provide informed consent for this (Appendix 5).

**Table 1. Data from medical journals, the lung cancer clinical database, the Civil Registration System and questionnaires**

| Variables | Data |
|---|---|
| *Medical journal, the lung cancer clinical database and the Civil Registration System* | |
| Treatment adherence 85% | Date of skipped treatments or delays if any, and dose administered |
| Lung cancer diagnosis | Histology, stage and performance status |
| Standard treatment initiated | Surgery, chemotherapy, radiotherapy, immunotherapy and medical treatment of side-effects |
| Vital status | Date of death or date last registered in live |
| Municipality rehabilitation | Referral status |
| Follow-up adherence | Date of non-response, if any |
| Comorbidities and medical treatment | Date of diagnosis of comorbidities and medical treatment for comorbidities, if any |
| Patient reported factors related to vulnerability (Appendix 9) | Activities of daily living, social support, health literacy, barriers for treatment and alcohol abuse |
| *Questionnaire* | |
| Demographics (baseline only) | Age, gender, partner, education, job |
| HRQoL | EORTC QLQ-C30[65] + lung QLQ-LC13[66] + 5Q-5D-5L[67] |
| Health behavior | Alcohol, smoking, physical training and nutrition |
| Self-activation/self-efficacy | 3 items |

EORTC QLQ-C30; The European Organization for Research and Treatment of Cancer Quality-of-life Questionnaire Core 30
Lung QLQ-LC13; Quality-of-life Questionnaire Lung Cancer 13
5Q-5D-5L; European Quality of life Questionnaire- 5 Dimensions - 5 Levels

# Appendix 2

## Feasibility study

In order to adjust the RCT procedure and the intervention we will conduct a feasibility study including 20 lung cancer patients. The first 20 patients recruited for the study will not be randomized, but will instead receive the intervention in order to adjust study and intervention procedures especially the feasibility of the inclusion procedure and the acceptability of intervention based on feedback from health professionals, researchers and patients. The feasibility study will be conducted at the Department of Clinical Oncology and Palliative Care at Zealand University Hospital in Roskilde. Data will be collected at baseline and 3 months, 6 months and 12 months after diagnosis as in the randomized study enabling continuous adjustments of the study and intervention procedures. Data from the pilot study will not be used in the analyses of the study effects. Participants in the pilot study are informed in the patient information that they will receive the intervention without randomization (Appendix 3a). If the pilot study leads to any changes of the intervention that will improve the acceptability of the intervention, an amendment will be submitted to the Ethical Committee.

## Process evaluation

We will conduct a process evaluation  of the implementation of the intervention for its delivery and mechanisms of impact based on the Medical Research Council guidance on process evaluations of complex interventions [68]. The process evaluation on the delivery of intervention will focus on fidelity (to what extend it was carried out as planned), dose (the amount and intensity of intervention), adaptation (any changes made to the intervention) and reach (to what extent we reached the intended population and the match between their needs and the intervention). The evaluation of the mechanisms of impact will focus on experiences of and interactions between the participants and the intervention and the context (any external influences). This evaluation will be performed by a qualitative researcher with on prior knowledge of the study, who will conduct participant-observations during training and patient sessions with subsequent qualitative semi-structured interviews with participants about their experience of the intervention component; however, only among participants who provide informed consent for this (Appendix 5). Finally,

focus group interviews with nurse navigators and physiotherapists will be conducted about their perceptions of the intervention (facilitators and barriers).

## Power considerations

The power calculation is based on a presumed improvement in 1-year survival of 13% corresponding to half of the effect in the study by Denis et al [55]. Assuming that patients in the control group have a 50% one-year overall survival and the intervention group have 63% one-year overall survival and a 15% withdrawal probability, then we have 80% power to detect a significant difference using a log-rank test if we have a total of 518 patients i.e. 259 per group. Therefore, a total of 538 patients will be included in the study (20 pilot patients and 518 patients in the randomized controlled trial). Table 2 shows sample size for different combinations of one-year survival and withdrawal probability. Assuming that an estimated 60% will be considered vulnerable (N=865) and a 50% response rate would mean that a total of 1,730 patients should be invited.

**Table 2. Required sample size for different combinations of one-year survival and withdrawal**

| One-year survival Intervention | One-year survival control | Withdrawal probability | N per group | N total |
|---|---|---|---|---|
| 0.55 | 0.50 | 0.10 | 1,679 | 3,358 |
| 0.55 | 0.50 | 0.15 | 1,777 | 3,554 |
| 0.55 | 0.50 | 0.20 | 1,888 | 3,776 |
| 0.60 | 0.50 | 0.10 | 417 | 834 |
| 0.60 | 0.50 | 0.15 | 441 | 882 |
| 0.60 | 0.50 | 0.20 | 469 | 938 |
| 0.63 | 0.50 | 0.10 | 244 | 488 |
| 0.63 | 0.50 | 0.15 | 259 | 518 |
| 0.63 | 0.50 | 0.20 | 275 | 550 |
| 0.65 | 0.50 | 0.10 | 182 | 364 |
| 0.65 | 0.50 | 0.15 | 193 | 386 |
| 0.65 | 0.50 | 0.20 | 205 | 410 |

## Statistical analyses

Descriptive statistics will be used to estimate the frequencies, means and standard deviations of the study variables. Analyses will be based on intention-to-treat with primary analyses testing the effect of the intervention on overall survival, and secondary analyses testing the effect on treatment adherence (85% compliance), physical symptom burden, psychological symptoms, HRQol and health behavior. Overall survival probabilities will be estimated by calculating survival time from the date of enrollment until date of death, date of withdrawal or end of follow-up. Cox proportional hazards models with time since randomization as the underlying time will be used to estimate the hazard ratio and 95% confidence intervals for overall survival associated with the intervention arm using the control arm as the reference group. Secondary outcomes will be evaluated by using mixed effect models. Additional analyses will include evaluating the impact of potential effect modifiers.

## Study period and budget

### *Study period*

The preparations for this study was initiated in March 2020 where we held a workshop for brain-storming ideas and establishing a national network of clinicians and researchers. We are currently conducting preparatory studies regarding the development of the screening instrument for vulnerability and the intervention manual describing the response algorithm for PRO symptoms, the content and format of the nurse sessions and the physical training component. The recruitment of patients is expected to begin in March 2021 after permission form the Ethical Committee has been obtained. The study phases and milestones are illustrated in the Gantt chart below.

# Appendix 2

| Task | Period | Year 1 | | | Year 2 | | | Year 3 | | | Year 4 | | | Year 5 | | |
|---|---|---|---|---|---|---|---|---|---|---|---|---|---|---|---|---|
| Months | | 1-4 | 5-8 | 9-12 | 1-4 | 5-8 | 9-12 | 1-4 | 5-8 | 9-12 | 1-4 | 5-8 | 9-12 | 1-4 | 5-8 | 9-12 |
| Study protocol and funding applications [a] | | ■ | ■ | ■ | | | | | | | | | | | | |
| Application for regional data protection boards and ethics board [b] | | ■ | ■ | ■ | | | | | | | | | | | | |
| Patient involvement in development of intervention and screening [c] | | | ■ | ■ | | | | | | | | | | | | |
| Pilot test of screening instrument [d] | | | ■ | ■ | | | | | | | | | | | | |
| Training of nurse navigators [e] | | | | | ■ | | | | | | | | | | | |
| Pilot test of intervention [f] | | | | | ■ | | | | | | | | | | | |
| Recruitment of patients starts in Roskilde [g] | | | | | ■ | ■ | ■ | ■ | ■ | | | | | | | |
| Recruitment in other centers [h] | | | | | | ■ | ■ | ■ | ■ | ■ | | | | | | |
| Intervention [i] | | | | | | ■ | ■ | ■ | ■ | ■ | ■ | ■ | ■ | | | |
| Data collection [j] | | | | | | ■ | ■ | ■ | ■ | ■ | ■ | ■ | ■ | | | |
| Analyses [k] | | | | | | | | | | | | ■ | ■ | ■ | | |
| Reporting of results [l] | | | | | | | | | | | | | ■ | ■ | ■ | ■ |

[a] Preparation of study protocol and invitation to all relevant departments in DK to participate in the study. We will apply for funding for the multicenter RCT.
[b] All five regional data protection boards in Denmark will be applied as well as the Regional Zealand Ethic Board.
[c] Patients (N=15) will be involved in the development of the screening questionnaire for vulnerability and in the intervention components using cognitive and qualitative semi-structured interviews.
[d] We will pilot test the screening questionnaire in 100 patients and obtain information on treatment adherence from medical records.
[e] Nurse navigators will receive a 5-day course in study procedures and intervention components including prehabilitation program, PRO monitoring and cognitive therapy strategies used in sessions with patients.
[f] We will pilot test the intervention in a feasibility study including 20 lung cancer patients at the Department of Clinical Oncology and Palliative Care at Zealand University Hospital in Roskilde.
[g] We have received funding for part of the multicenter RCT and plan to begin the recruitment of patients in Roskilde.
[h] The recruitment of patients will continue in other centers when additional funding has been obtained.
[i] The intervention will include nurse-navigation, exercise training and PRO monitoring.
[j] Data is collected by questionnaires at baseline, 3, 6 and 12 months after diagnosis and consecutively from the lung cancer clinical database and individual medical records.
[k] Statistician Elisabeth W Andersen will perform the analyses in collaboration with the project group.
[l] Results will be presented and discussed in peer-reviewed journals and at international conferences.

# Appendix 2

## *Budget*

The budget in Table 3 defines estimated overall expenses for the multicenter RCT study. We have obtained partly funding for our study coordinator postdoc Rikke Langballe and nurse navigators in Roskilde as part of a work package within COMPAS. We are applying for further funding to cover expenses for recruitment of patients and nurse navigators at the additional 8 study centers (Table 3).

**Table 3. Budget for the multicenter RCT study**

| Expenses | Period | Year 1 | Year 2 | Year 3 | Year 4 | Year 5 | Total |
|---|---|---|---|---|---|---|---|
| Study coordinator[a] | | 652.000 | 652.000 | 652.000 | 652.000 | 326.000 | 2.934.000 |
| Nurse navigator[b] | | 158.437 | 975.854 | 975.854 | 487.927 | 0 | 2.598.072 |
| Development of intervention and education program[c] | | 278.000 | 0 | 0 | 0 | 0 | 278.000 |
| Recruitment[d] | | 80.000 | 518.400 | 259.200 | 0 | 0 | 857.600 |
| Physiotherapist[e] | | 0 | 1.092.933 | 1.092.933 | 0 | 0 | 2.185.867 |
| Statistician[f] | | 50.000 | 0 | 50.000 | 75.000 | 75.000 | 250.000 |
| Student[g] | | 57.600 | 57.600 | 57.600 | 57.600 | 57.600 | 288.000 |
| Running costs[h] | | 130.000 | 60.000 | 60.000 | 60.000 | 60.000 | 370.000 |
| Own financing[i] | | 0 | 0 | 0 | 0 | 0 | 0 |
| Total | | 1.406.037 | 3.356.788 | 3.147.588 | 1.332.527 | 518.600 | 9.761.539 |
| Overhead (5%) | | 70.302 | 167.839 | 157.379 | 66.626 | 25.930 | 488.077 |
| Amount incl. overhead | | 1.476.339 | 3.524.627 | 3.304.967 | 1.399.153 | 544.530 | 10.249.616 |

[a] Salary for project coordinator post doc Rikke Langballe for 4,5 years.
[b] Salary for one part-time (average 10 hours per week) nurse navigator for 2,5 years (year 2+3+4) at 12 study centers. Expenses year 1 include 5 days salary for 12 nurses and accommodation during the training course as well as travel expenses.
[c] Two months' nurse salary for nurse Birgitte Mertz for help with developing the intervention and training the nurse navigators from each study center and funding from the Danish Comprehensive Cancer Center to cover development of a national network of clinicians and researchers.
[d] Clinical research units or other local establishments will perform the recruitment of patients for the pilot study of the screening questionnaire in year 1 and for the RCT study in year 2+3 (1.5 year).
[e] Salary for physiotherapists who will supervise patients 3 x weekly for 12 weeks.
[f] Salary for statistician Elisabeth W Andersen for 4 months in the analyses process.
[g] Student salary for 8 hours per week at 150 kr. per hour for follow-up and data entry.
[h] Running costs include: Open access publication (25.000 kr), conferences (40.000 kr) questionnaire design (20.000 kr), brochure design & print (30.000 kr), tablets (12 x 4000 kr=48.000 kr), video guide for study information (30.000 kr), letters (envelops: 1.500 kr, return letter account at Post Denmark for 3 years: 13.932 kr, 26 x PRO questionnaires for 20% participants: 54.080 kr, 3 x baseline and follow-up questionnaires for 20% participants: 9.984 kr, 1 x reminder to 259 participants: 2.590 kr), registry data (40.000 kr), travel expenses between study centers during trial (25.000 kr) and meetings (2 x 15.000 kr=30.000 kr).
[i] Research assistant contributes with ½ months' salary per year.

Appendix 2

## Ethical considerations

The study will be approved by and follow the requirements from The Regional Committee on Health Research Ethics in Region Zealand. The study will follow the CONSORT recommendations for randomized controlled trials of non-pharmacological treatment [69] and will be made public at ClinicalTrials.gov before inclusion of the first participant. Participation will be voluntary and patients will receive written and verbal information about the study and sign written informed consent before study participation. Participants will have the right to withdraw from the study at any time without giving any reason and with no consequences for continued treatment. Participants will not be restricted from any activities or treatments outside the study. "The Danish Patient Insurance" covers the participants in this study.

Patients invited to the Navigate trial are vulnerable, but studies suggest that participants in clinical trials have better [70] or similar outcomes [71] as non-participants, indicating no increased harm associated with participation. We cannot rule out that weekly collection of physical and psychological symptoms during treatment may lead to increased risk for uncertainty and anxiety [72] or that participation in the exercise program will lead to minor adverse-effects such as muscle soreness. All participants in the study are instructed to report if they experience any side effects, risks or harms associated with study participation. There are no known circumstances that may lead to disruption of this study or that participants will be excluded from study participation.

Data collected for this study will be securely stored according to the General Data Protection Regulation (GDPR) requirements at The Danish Cancer Society Research Center and the Data Protection Art will be followed.

## Dissemination of study results

We plan to publish at least three scientific papers directly on the study results as well as five papers related to the trial in peer-reviewed journals. The study results will be presented at international conferences and at the COMPAS website. Moreover, we plan to communicate the

results to the public in a video format at social media platforms and by releasing newsletters through the communication department at the Danish Cancer Society.

## Risk mitigation plan

The overarching challenges related to the study include recruitment of patients and attrition during the exercise program. We plan to address these challenges by applying a high degree of patient involvement in the development phase and by assigning the recruitment of patients to trained research nurses from clinical research units at each participating department.

## Project organization

Professor, MD Susanne Dalton, chief physician Mads Nordahl Svendsen, senior researcher, Ph.D. Pernille Bidstrup and associated professor, MD, Erik Jacobsen will be the principle investigators of the project. The daily project coordinator will be postdoc Rikke Langballe, who will coordinate the development and implementation of the intervention and will be responsible for planning the statistical analyses and reporting of results in international peer-reviewed journals and at conferences through supervision of the principle investigators. The project group further includes project coordinator Randi Valbjørn Karlsen, managing head nurse Helle Gert, head nurse Camilla Kjærulff, postdoc Elizabeth Emilie Rosted and chief physician Jeanette Ehlers. International collaboration partners include Karen Freund and Amy Michelle LeClair, Tufts University School of Medicine. Moreover, professor Brendan McCormack, head of the Division of Nursing at Queen Margaret University in Edinburgh, and professor in nursing at Zealand University Hospital, will have a role as an external advisor for the study. We will not exchange any data with our international collaborators as the collaboration will concern development of the intervention and reporting of aggregated results in peer-reviewed journals. Other close national collaboration partners include professor and head of the Danish National Center for Lung Cancer Research, Ole Hilberg who will contribute scientifically and with patient recruitment. Moreover, professor, physiotherapist Søren Thorgaard Skou and physiotherapist, PhD student Lars Bo Jørgensen are

collaboration partners in developing the training program. We will furthermore establish a multicenter consortium with nurses and physicians from each participating study center. The project is supported by the Danish Comprehensive Cancer Center (DCCC), as social inequality in cancer presents a great challenge that calls for national collaboration.

## Perspectives

Results from the NAVIGATE trial hold great potential in improving symptom management and supportive care to vulnerable lung cancer patients with fragile social support, low health literacy and complex needs. The primary success criteria is that patients in the intervention group experience clinically relevant improvements in survival and symptoms with at least 10% reduction in one of the key physical symptoms. The ultimate success criteria will be implementation of the intervention at lung cancer clinics in Denmark. By applying a high degree of patient involvement in the development phase and evaluating the intervention in a national multicenter setting, we hope to optimize the potential for implementing the NAVIGATE intervention if the effects are positive. The NAVIGATE intervention has the potential to make an international contribution to lung cancer care by providing clinicians with a new comprehensive model of care targeting vulnerable patients. Of paramount importance, we hope to give vulnerable lung cancer patients a greater possibility to achieve the same treatment outcomes as the more resourceful patients and thereby taking the first steps towards reducing inequality in our health care system.

Appendix 2

## References


1. Dalton SO, Steding-Jessen M, Engholm G, Schuz J, Olsen JH. Social inequality and incidence of and survival from lung cancer in a population-based study in Denmark, 1994-2003. *European journal of cancer (Oxford, England : 1990).* 2008;44(14):1989-1995.
2. Sidorchuk A, Agardh EE, Aremu O, Hallqvist J, Allebeck P, Moradi T. Socioeconomic differences in lung cancer incidence: a systematic review and meta-analysis. *Cancer Causes Control.* 2009;20(4):459-471.
3. Bray F, Ferlay J, Soerjomataram I, Siegel RL, Torre LA, Jemal A. Global cancer statistics 2018: GLOBOCAN estimates of incidence and mortality worldwide for 36 cancers in 185 countries. *CA Cancer J Clin.* 2018;68(6):394-424.
4. Weller DP, Peake MD, Field JK. Presentation of lung cancer in primary care. *npj Primary Care Respiratory Medicine.* 2019;29(1).
5. *Danish Lung Cancer Group (DLCG). Annual Report* 2018.
6. Mellemgaard A, Andersen JL, Seppo L. Medicinsk behandling af lungecancer. *Ugeskr Læger.* 2018;180:2088-2091.
7. Forrest LF, Adams J, Wareham H, Rubin G, White M. Socioeconomic inequalities in lung cancer treatment: systematic review and meta-analysis. *PLoS Med.* 2013;10(2):e1001376.
8. Kaergaard Starr L, Osler M, Steding-Jessen M, et al. Socioeconomic position and surgery for early-stage non-small-cell lung cancer: A population-based study in Denmark. *Lung Cancer.* 2013;79(3):262-269.
9. Dalton SO, Steding-Jessen M, Jakobsen E, et al. Socioeconomic position and survival after lung cancer: Influence of stage, treatment and comorbidity among Danish patients with lung cancer diagnosed in 2004-2010. *Acta Oncol.* 2015;54(5):797-804.
10. Berglund A, Holmberg L, Tishelman C, Wagenius G, Eaker S, Lambe M. Social inequalities in non-small cell lung cancer management and survival: a population-based study in central Sweden. *Thorax.* 2010;65(4):327-333.
11. Tendler S, Holmqvist M, Wagenius G, Lewensohn R, Lambe M, De Petris L. Educational level, management and outcomes in small-cell lung cancer (SCLC): A population-based cohort study. *Lung Cancer.* 2020;139:111-117.
12. Willen L, Berglund A, Bergstrom S, et al. Educational level and management and outcomes in non-small cell lung cancer. A nationwide population-based study. *Lung Cancer.* 2019;131:40-46.
13. Halgren MO, Kjær TK, Dalton SO. *Hvidbog - Social ulighed i kræft i Danmark.* 2019.
14. Fowler H, Belot A, Ellis L, et al. Comorbidity prevalence among cancer patients: a population-based cohort study of four cancers. *BMC Cancer.* 2020;20(1):2.
15. Zeng L, Yu X, Yu T, Xiao J, Huang Y. Interventions for smoking cessation in people diagnosed with lung cancer. *The Cochrane database of systematic reviews.* 2019;6:Cd011751.
16. Christensen NL, Lokke A, Dalton SO, Christensen J, Rasmussen TR. Smoking, alcohol, and nutritional status in relation to one-year mortality in Danish stage I lung cancer patients. *Lung Cancer.* 2018;124:40-44.
17. Evangelista LS, Sarna L, Brecht ML, Padilla G, Chen J. Health perceptions and risk behaviors of lung cancer survivors. *Heart Lung.* 2003;32(2):131-139.

18. Cooley ME, Finn KT, Wang Q, et al. Health behaviors, readiness to change, and interest in health promotion programs among smokers with lung cancer and their family members: a pilot study. *Cancer Nurs.* 2013;36(2):145-154.
19. Tsao AS, Liu D, Lee JJ, Spitz M, Hong WK. Smoking affects treatment outcome in patients with advanced nonsmall cell lung cancer. *Cancer.* 2006;106(11):2428-2436.
20. Wang S, Wong ML, Hamilton N, Davoren JB, Jahan TM, Walter LC. Impact of age and comorbidity on non-small-cell lung cancer treatment in older veterans. *J Clin Oncol.* 2012;30(13):1447-1455.
21. Ajzen I. The Theory of Planned Behavior. *Organisational Behaviour and Human Decision Processes.* 1991;50:179-211.
22. Sharf BF, Stelljes LA, Gordon HS. 'A little bitty spot and I'm a big man': patients' perspectives on refusing diagnosis or treatment for lung cancer. *Psychooncology.* 2005;14(8):636-646.
23. Keeley B, Wright L, Condit CM. Functions of health fatalism: fatalistic talk as face saving, uncertainty management, stress relief and sense making. *Sociol Health Illn.* 2009;31(5):734-747.
24. Kaplan AL, Crespi CM, Saucedo JD, Connor SE, Litwin MS, Saigal CS. Decisional conflict in economically disadvantaged men with newly diagnosed prostate cancer: baseline results from a shared decision-making trial. *Cancer.* 2014;120(17):2721-2727.
25. Koay K, Schofield P, Jefford M. Importance of health literacy in oncology. *Asia Pac J Clin Oncol.* 2012;8(1):14-23.
26. Chabowski M, Polanski J, Jankowska-Polańska B, Rosińczuk J, Szymanska-Chabowska A. Quality of life of patients with lung cancer. *OncoTargets and Therapy.* 2016:1023.
27. Iyer S, Roughley A, Rider A, Taylor-Stokes G. The symptom burden of non-small cell lung cancer in the USA: a real-world cross-sectional study. *Support Care Cancer.* 2014;22(1):181-187.
28. Basch E, Deal AM, Kris MG, et al. Symptom Monitoring With Patient-Reported Outcomes During Routine Cancer Treatment: A Randomized Controlled Trial. *J Clin Oncol.* 2016;34(6):557-565.
29. Hansen NHG, Levinsen AKG, Carlsen S. *Socioeconomic position and patient-related factors and their association with completion of first-line treatment among lung cancer patients*: Department of Public Health, University of Copenhagen; 2019.
30. Kyte K, Ekstedt M, Rustoen T, Oksholm T. Longing to get back on track: Patients' experiences and supportive care needs after lung cancer surgery. *J Clin Nurs.* 2019;28(9-10):1546-1554.
31. Koay K, Schofield P, Gough K, et al. Suboptimal health literacy in patients with lung cancer or head and neck cancer. *Support Care Cancer.* 2013;21(8):2237-2245.
32. Freund KM, Battaglia TA, Calhoun E, et al. Impact of patient navigation on timely cancer care: the Patient Navigation Research Program. *Journal of the National Cancer Institute.* 2014;106(6):dju115.
33. Rodday AM, Parsons SK, Snyder F, et al. Impact of patient navigation in eliminating economic disparities in cancer care. *Cancer.* 2015;121(22):4025-4034.
34. Doerfler-Evans RE. Shifting paradigms continued-the emergence and the role of nurse navigator. *J Thorac Dis.* 2016;8(Suppl 6):S498-500.

35. Zibrik K, Laskin J, Ho C. Integration of a nurse navigator into the triage process for patients with non-small-cell lung cancer: creating systematic improvements in patient care. *Curr Oncol.* 2016;23(3):e280-283.
36. Hunnibell LS, Rose MG, Connery DM, et al. Using nurse navigation to improve timeliness of lung cancer care at a veterans hospital. *Clin J Oncol Nurs.* 2012;16(1):29-36.
37. Zibrik K, Laskin J, Ho C. Implementation of a Lung Cancer Nurse Navigator Enhances Patient Care and Delivery of Systemic Therapy at the British Columbia Cancer Agency, Vancouver. *J Oncol Pract.* 2016;12(3):e344-349.
38. Schofield P, Ugalde A, Gough K, et al. A tailored, supportive care intervention using systematic assessment designed for people with inoperable lung cancer: a randomised controlled trial. *Psychooncology.* 2013;22(11):2445-2453.
39. Ludman EJ, McCorkle R, Bowles EA, et al. Do depressed newly diagnosed cancer patients differentially benefit from nurse navigation? *Gen Hosp Psychiatry.* 2015;37(3):236-239.
40. Wagner EH, Ludman EJ, Aiello Bowles EJ, et al. Nurse Navigators in Early Cancer Care: A Randomized, Controlled Trial. *Journal of Clinical Oncology.* 2014;32(1):12-18.
41. Skrutkowski M, Saucier A, Eades M, et al. Impact of a pivot nurse in oncology on patients with lung or breast cancer: symptom distress, fatigue, quality of life, and use of healthcare resources. *Oncol Nurs Forum.* 2008;35(6):948-954.
42. Mertz BG, Dunn-Henriksen AK, Kroman N, et al. The effects of individually tailored nurse navigation for patients with newly diagnosed breast cancer: a randomized pilot study. *Acta Oncologica.* 2017;56(12):1682-1689.
43. Envold Bidstrup P, Mertz BG, Kroman N, et al. Tailored nurse navigation for women treated for breast cancer: Design and rationale for a pilot randomized controlled trial. *Acta Oncologica.* 2016;55(9-10):1239-1243.
44. Peddle-McIntyre CJ, Singh F, Thomas R, Newton RU, Galvao DA, Cavalheri V. Exercise training for advanced lung cancer. *The Cochrane database of systematic reviews.* 2019;2:Cd012685.
45. Rosero ID, Ramirez-Velez R, Lucia A, et al. Systematic Review and Meta-Analysis of Randomized, Controlled Trials on Preoperative Physical Exercise Interventions in Patients with Non-Small-Cell Lung Cancer. *Cancers (Basel).* 2019;11(7).
46. Liu Z, Qiu T, Pei L, et al. Two-Week Multimodal Prehabilitation Program Improves Perioperative Functional Capability in Patients Undergoing Thoracoscopic Lobectomy for Lung Cancer: A Randomized Controlled Trial. *Anesth Analg.* 2019.
47. Giles AE, Srinathan SK. Prehabilitation prior to lung cancer surgery: a small step forward. *J Thorac Dis.* 2019;11(12):5664-5665.
48. Bhatia C, Kayser B. Preoperative high-intensity interval training is effective and safe in deconditioned patients with lung cancer: A randomized clinical trial. *J Rehabil Med.* 2019;51(9):712-718.
49. Mujcic A, Blankers M, Bommele J, et al. *Psychooncology.* 2020;29(1):49-60.
50. Kock L, Brown J, Hiscock R, Tattan-Birch H, Smith C, Shahab L. Individual-level behavioural smoking cessation interventions tailored for disadvantaged socioeconomic position: a systematic review and meta-regression. *Lancet Public Health.* 2019;4(12):e628-e644.
51. Aveyard P, Begh R, Parsons A, West R. Brief opportunistic smoking cessation interventions: a systematic review and meta-analysis to compare advice to quit and offer of assistance. *Addiction.* 2012;107(6):1066-1073.

52. Stuart H, Mejding K. *Very Brief Advice - Meget kort rådgivning om rygestop.* Copenhagen2017.
53. Kotronoulas G, Kearney N, Maguire R, et al. What is the value of the routine use of patient-reported outcome measures toward improvement of patient outcomes, processes of care, and health service outcomes in cancer care? A systematic review of controlled trials. *J Clin Oncol.* 2014;32(14):1480-1501.
54. Bouazza YB, Chiairi I, El Kharbouchi O, et al. Patient-reported outcome measures (PROMs) in the management of lung cancer: A systematic review. *Lung Cancer.* 2017;113:140-151.
55. Denis F, Lethrosne C, Pourel N, et al. Randomized Trial Comparing a Web-Mediated Follow-up With Routine Surveillance in Lung Cancer Patients. *Journal of the National Cancer Institute.* 2017;109(9).
56. Denis F, Basch E, Septans AL, et al. Two-Year Survival Comparing Web-Based Symptom Monitoring vs Routine Surveillance Following Treatment for Lung Cancer. *Jama.* 2019;321(3):306-307.
57. Basch E, Deal AM, Dueck AC, et al. Overall Survival Results of a Trial Assessing Patient-Reported Outcomes for Symptom Monitoring During Routine Cancer Treatment. *JAMA.* 2017;318(2):197-198.
58. O'Cathain A, Croot L, Duncan E, et al. Guidance on how to develop complex interventions to improve health and healthcare. *BMJ open.* 2019;9(8):e029954.
59. Spencer JC, Wheeler SB. A systematic review of Motivational Interviewing interventions in cancer patients and survivors. *Patient education and counseling.* 2016;99(7):1099-1105.
60. Basch E, Deal AM, Kris MG, et al. Symptom Monitoring With Patient-Reported Outcomes During Routine Cancer Treatment: A Randomized Controlled Trial. *Journal of clinical oncology : official journal of the American Society of Clinical Oncology.* 2016.
61. Bidstrup PE, Mertz BG, Dalton SO, et al. Accuracy of the Danish version of the 'distress thermometer'. *Psychooncology.* 2012;21(4):436-443.
62. Carroll C, Patterson M, Wood S, Booth A, Rick J, Balain S. A conceptual framework for implementation fidelity. *Implementation science : IS.* 2007;2:40.
63. Schmidt M, Schmidt SA, Sandegaard JL, Ehrenstein V, Pedersen L, Sorensen HT. The Danish National Patient Registry: a review of content, data quality, and research potential. *Clin Epidemiol.* 2015;7:449-490.
64. Petersson F, Baadsgaard M, Thygesen LC. Danish registers on personal labour market affiliation. *Scand J Public Health.* 2011;39(7 Suppl):95-98.
65. Aaronson NK, Ahmedzai S, Bergman B, et al. The European Organization for Research and Treatment of Cancer QLQ-C30: a quality-of-life instrument for use in international clinical trials in oncology. *Journal of the National Cancer Institute.* 1993;85(5):365-376.
66. Bergman B, Aaronson NK, Ahmedzai S, Kaasa S, Sullivan M. The EORTC QLQ-LC13: a modular supplement to the EORTC Core Quality of Life Questionnaire (QLQ-C30) for use in lung cancer clinical trials. EORTC Study Group on Quality of Life. *European journal of cancer (Oxford, England : 1990).* 1994;30a(5):635-642.
67. Rabin R, de Charro F. EQ-5D: a measure of health status from the EuroQol Group. *Annals of medicine.* 2001;33(5):337-343.
68. Craig P, Dieppe P, Macintyre S, Michie S, Nazareth I, Petticrew M. Developing and evaluating complex interventions: the new Medical Research Council guidance. *Int J Nurs Stud.* 2013;50(5):587-592.

69. Boutron I, Moher D, Altman DG, Schulz KF, Ravaud P. Extending the CONSORT statement to randomized trials of nonpharmacologic treatment: explanation and elaboration. *AnnInternMed.* 2008;148(4):295-309.
70. Davis S, Wright PW, Schulman SF, et al. Participants in prospective, randomized clinical trials for resected non-small cell lung cancer have improved survival compared with nonparticipants in such trials. *Cancer.* 1985;56(7):1710-1718.
71. Vist GE, Bryant D, Somerville L, Birminghem T, Oxman AD. Outcomes of patients who participate in randomized controlled trials compared to similar patients receiving similar interventions who do not participate. *The Cochrane database of systematic reviews.* 2008(3):Mr000009.
72. Nielsen AS, Kidholm K, Kayser L. Patients' reasons for non-use of digital patient-reported outcome concepts: A scoping review. *Health informatics journal.* 2020:1460458220942649.
